# Serious Food Aspect

**Published:** 1920-03-06

**Authors:** 


### Serious Food Aspect.

### Urgent Need of Closer Inspection.

Public attention concerning food supplies, which by the necessities of the case flits from milk to rice, from rice to bread, or from bread to meat, as the supplies and prices themselves vary, has this week been drawn largely towards the last couple—namely, bread and meat. One question that glowers at us is whether we are soon to pay a shilling for the loaf. On the other hand, Sir Auckland Geddes—not, however, a consistently reliable guide—holds out hope of a reduction in the price of mutton.

But what is more serious than the price of mutton and of meat in general is the question of its quality, and there have been reports of bad meat in the Metropolitan area. There is no doubt that this state of things does not apply only to the London districts, and it is a question that demands urgent consideration. Far too little notice has been taken of the serious statements on the subject contained in the last report of the medical department of the Local Government Board, which demonstrates conclusively what little protection the public enjoys from dangers due to the consumption of meat derived from diseased animals. From the public health standpoint the most serious aspect of the lack of systematic meat inspection is the facility which is afforded for the unchecked disposal of cow carcases for human food. A very considerable proportion of the cows slaughtered annually in this country are seriously affected with tuberculosis, and under conditions which normally exist in England and Wales it is safe to say that only a small proportion of these cows ever comes under inspection at the time of their slaughter. It is doubtful whether any large proportion of the meat derived from them subsequently comes under inspection in the town markets and shops or in rural hawkers' vans, but in any case it cannot be too strongly emphasised that whatever inspection may be given to meat of this or any other class after it has been dressed and cut into portions for sale it is generally quite incapable of detecting whether or not the meat was derived from an animal in a diseased condition.

There is only one method of ensuring the freedom of meat from disease, and that is by careful examination of the carcase at the time of slaughter, when all viscera are available for inspection. The only practicable means by which this can be brought about is by requiring all animals intended for food purposes to be brought to a public abattoir and by providing skilled inspectors in sufficient numbers to inspect thoroughly both before and after slaughter every animal brought there. This would result in the closing of all private slaughterhouses as such, and in order to be of practical advantage to public health the requirements would need to operate in town and country alike. Their application to urban areas alone would not be sufficient, as this would merely accentuate past evils by driving the trade in diseased animals exclusively into rural areas where there is no inspection at all. It may take some time to establish the system of slaughtering and inspection in public abattoirs generally throughout the country. If in the meantime, however, urban authorities, which had set up the system within their areas, were placed in a position to require that all meat brought into their districts from outside areas should be marked as having been killed and inspected in a recognised public abattoir this would provide a useful stimulus to the erection of abattoirs in meat-producing rural areas.

The position in regard to this matter is from the public health point of view most unsatisfactory. The difficulties in the way of dealing with it in the past have been very great, but the opposition to public abattoirs which has hitherto been encountered from butchers may possibly be modified in future on account of the altered conditions which have resulted under the Ministry of Food's restrictions as to slaughtering. These have in many districts accustomed butchers to centralised slaughtering, and may help to overcome their prejudice against the establishment of permanent central abattoirs. In any case, for one reason and another, the number of private slaughterhouses which will be in use after control of the meat trade has been removed will be less than before control was established. For these reasons (says the report) the present seems to be a favourable opportunity for the promotion of public abattoirs on as large a scale as possible and the compulsory closing of private slaughterhouses.

Why, therefore, is not authority at once given to local bodies to safeguard themselves on the lines suggested? That seems the obvious first step, and the exercise of such powers by progressive local authorities would of itself gradually lead up to a universal system of efficient meat inspection.

